# Chidamide Reverses Fluzoparib Resistance in Triple-Negative Breast Cancer Cells

**DOI:** 10.3389/fonc.2022.819714

**Published:** 2022-02-18

**Authors:** Xinyang Li, Xiang Yuan, Ziming Wang, Jing Li, Zhiwei Liu, Yukun Wang, Limin Wei, Yuanpei Li, Xinshuai Wang

**Affiliations:** ^1^ Henan Key Laboratory of Cancer Epigenetics, Cancer Hospital, The First Affiliated Hospital, College of Clinical Medicine, Medical College of Henan University of Science and Technology, Luoyang, China; ^2^ Department of Internal Medicine, UC Davis Comprehensive Cancer Center, University of California, Davis, Sacramento, CA, United States

**Keywords:** triple-negative breast cancer, PARP inhibitor resistance, drug resistance reversal, fluzoparib, chidamide

## Abstract

Poly (ADP-ribose) polymerase inhibitor (PARPi) resistance is a new challenge for antitumor therapy. The purpose of this study was to investigate the reversal effects of chidamide on fluzoparib resistance, a PARPi, and its mechanism of action. A fluzoparib-resistant triple-negative breast cancer (TNBC) cell line was constructed, and the effects of chidamide and fluzoparib on drug-resistant cells were studied *in vitro* and *in vivo*. The effects of these drugs on cell proliferation, migration, invasiveness, the cell cycle, and apoptosis were detected using an MTT assay, wound-healing and transwell invasion assays, and flow cytometry. Bioinformatics was used to identify hub drug resistance genes and Western blots were used to assess the expression of PARP, RAD51, MRE11, cleaved Caspase9, and P-CDK1. Xenograft models were established to analyze the effects of these drugs on nude mice. *In vivo* results showed that chidamide combined with fluzoparib significantly inhibited the proliferation, migration, and invasiveness of drug-resistant cells and restored fluzoparib sensitivity to drug-resistant cells. The combination of chidamide and fluzoparib significantly inhibited the expression of the hub drug resistance genes RAD51 and MRE11, arrested the cell cycle at the G2/M phase, and induced cell apoptosis. The findings of this work show that chidamide combined with fluzoparib has good antineoplastic activity and reverses TNBC cell resistance to fluzoparil by reducing the expression levels of RAD51 and MRE11.

## Introduction

Breast cancer, the most common malignant solid tumor, is the leading cause of cancer deaths among women worldwide, with approximately 2.1 million new cases in 2020 alone (1). Triple-negative breast cancer (TNBC), a subtype of breast cancer that lacks the expression of hormone receptors (estrogen receptor or progesterone receptor) and human epidermal growth factor receptor 2, has a higher degree of virulence and is resistant to various chemotherapeutics and targeted medicine, making it challenging to treat (2, 3). Molecular-targeted precision therapy and predictive biomarkers associated with the diagnosis and treatment of TNBC are needed to comprehensively treat this malignancy.

Recent research in the field of DNA damage repair has shown that poly (ADP-ribose) polymerase inhibitors (PARPi) that involve the synthetic lethal approach have achieved satisfactory effects and promising prospects in the treatment of various cancers (4–6). PARPi traditionally exert antitumor effects by trapping PARP on DNA, causing DNA replication forks to collapse, disrupting cell mitosis, and inducing cell death, although chemoresistance to PARPi has been reported (7, 8). The mechanisms of PARPi resistance primarily include: (1) restoration of BRCA function or the abnormal expression of DNA repair proteins, leading to the restoration of the homologous recombination repair pathway (9–12); (2) deletion of PTIP, EZH2, and MUS81 expression or increased miR-493-5p expression, leading to stability of the replication fork (13–15); (3) mutations in PARP1 and PARG (16, 17); (4) creation of P-glycoprotein pumps and ATP-binding cassette drug transporters that increase drug outflow (18, 19); and (5) mir-622 overexpression, which inhibits nonhomologous end joining (20). Overcoming PARPi resistance is necessary to permit adequate PARPi antitumor therapy.

The identification of histone deacetylase (HDAC) as a new anticancer therapeutic target has added a new target for novel therapies. HDACs are involved in breast cancer tumorigenesis by regulating the genes of cell cycle factors, differentiation factors, and apoptotic factors (21–23). HDACs can also enhance genes related to angiogenesis, cell invasion, and migration and immune regulation to promote cancer development, such as vascular endothelial growth factor (VEGF), endothelial nitric oxide synthase (eNOS), HIF-1α, major histocompatibility complex (MHC), and human leukocyte antigens (HLA) (24–29). As a multilayer regulatory protein, HDAC can also affect DNA damage repair by regulating the expression of DNA damage repair-related genes and enhancing the activity of the DNA repair protein complex (30, 31). Most importantly, HDAC inhibitors (HDACi) have exhibited surprising antitumor effects.

Prior works have confirmed that HDACi combined with PARPi has a significant antitumor effect on TNBC cells (32, 33). In addition, HDACi can overcome gemcitabine, tamoxifen, and trastuzumab resistance (34–36). However, no studies on HDACi overcoming PARPi resistance are available. Therefore, based on the multifaceted antitumor activity of HDACi, we hypothesized that chidamide (HDACi) would reverse fluzoparib (PARPi) resistance. We investigated the mechanism behind HDACi reversal of PARPi resistance in breast cancer cells in the present work by constructing fluzoparib-resistant breast cancer cell lines. We demonstrate here that effective antitumor activity can be restored if fluzoparib is combined with chidamide.

## Materials and Methods

### Cell Culture

HCC1937 and MDA-MB-468 triple-negative breast cancer cell line were purchased from the China Center for Type Culture Collection (CCATCC, China) and cultured according to the instructions provided by the manufacturers. The fluzoparib-resistant cell lines HCC1937-FR and MDA-MB-468-FR were established at our institution.

### Chemicals and Antibodies

Fluzoparib is a PARP inhibitor and chidamide is a HDAC inhibitor. PARP, RAD51, MRE11, cleaved Caspase9, GAPDH, and P-CDK1 antibodies were obtained from Abcam Trading Co., Ltd. (Shanghai, China).

### Establishment of Drug-Resistant Cell Lines

The fluzoparib-resistant cell lines HCC1937-FR and MDA-MB-468-FR were constructed based on increasing drug concentration. HCC1937 and MDA-MB-468 cells in the logarithmic growth phase were cultured in complete medium at final fluzoparib concentrations of 2 and 5 μg/ml. After the cells were incubated for 2 days or when cell death reached 50%, the drug-containing medium was removed and the culture medium was passed 3 times or more with drug-free fresh medium. After waiting for the cell state to gradually recover and permit stable passage, the same drug concentration was used again 3 times, with increased drug concentration according to the cell growth. This strategy yielded the fluzoparib-resistant cell lines HCC1937-FR and MDA-MB-468-FR.

### Identification and Biological Process Analysis of Differentially Expressed Genes

High-throughput sequencing was used to perform whole-transcriptome sequencing of HCC1937 and HCC1937-FR cells in order to identify differentially expressed genes (DEGs) between parental and drug-resistant cells. Using differential expression analysis, genes with a *p*-value <0.05 and a log2FC >1 or <1 were considered DEGs. The biological processes of the enrichment analysis of DEGs were identified using the STRING database (https://string-db.org/). Biologic process analysis results were visualized using the ggplot2 package (version 1.26.0). DEGs related to DNA damage repair were selected for further analysis.

### Identification of Hub Drug Resistance Genes

DEGs related to DNA damage repair were uploaded to the STRING database in order to obtain the protein–protein interaction network and analyzed visually with Cytoscape software. The top 6 genes with the highest degree of gene association degree were labeled hub drug resistance genes. Based on the gene expression of the 6 hub drug resistance genes, histograms were created and genes of interest were selected for further analysis.

### Experimental Efficacy Studies

Experiments regarding the biological function of drug-resistant cells included parental (HCC1937 and MDA-MB-468) and drug-resistant cell lines (HCC1937-FR and MDA-MB-468-FR). Drug efficacy studies using the drug-resistant cell lines utilized PBS, fluzoparib (30 μg/ml), chidamide (3 or 6 μg/ml), and the combination of the two drugs (fluzoparib 30 μg/ml + chidamide 3 or 6 μg/ml).

### Cell Viability Assay

An MTT assay was used to evaluate cell viability. Cells were seeded in 96-well plates at a density of 3–5 × 10^3^ cells/well for 24 h, and then treated with the experimental drugs for 48 h according to their experimental group. The cell viability of each well was assessed using a microplate reader (Bio-Tek, Norcross, GA, USA), which measured the absorbance of each well at 570 nm. The mean IC_50_ value of the cells in each experimental group was computed using SPSS. The resistance index was defined as IC_50_ of drug-resistant cells/IC_50_ of parental cells.

### Wound-Healing Assay

The migration ability of drug-resistant cells and drug-treated cells was analyzed using a wound-healing assay. Cells were plated in 6-well plastic culture plates at a density of 5 × 10^3^ cells/well in culture medium until they reached 90% confluence. Either drug-containing or drug-free serum-free medium was added to each Petri well according to the experimental group and observed for 24 h. Cell migration was recorded at 0 and 24 h, and ImageJ software (NIH, Bethesda, MA, USA) was used to quantitatively analyze the degree of cell migration in the different experimental groups.

### Transwell Invasion Assays

Cell invasion ability was assessed using Transwell invasion assays. Transwell chambers coated with Matrigel with a bottom membrane aperture size of 8 μm (Sigma-Aldrich, St. Louis, MO, USA) were used to measure cell invasiveness. A total of 200 ml of drug-resistant or parental cells was resuspended in serum-free culture medium with PBS or an experimental drug for 24 h. After washing, fixing, and staining, 10 visual fields were randomly selected and a cell count under a ×100 magnification optical microscope was performed using ImageJ software.

### Cell Cycle Analysis

For the cell cycle arrest assay, cells were starved in 6-well plates for 24 h before treatment. Cells were treated with PBS or drug culture medium according to experimental grouping for 24 h. Processed cells were then scraped with PBS, fixed with 70% precooled ethanol for 1 h then washed again and conducted with RNase I for 30 min. The cells treated with PI staining at 4°C for 30 min were then measured using a BD FACS caliber.

### Apoptosis Analysis

An Annexin V-FITC/PI apoptosis detection kit was used to measure apoptosis rate. Cells were seeded into 6-well plates and exposed to the experimental drug for 48 h. A BD FACS caliber was used to detect cell apoptosis using the manufacturer’s instructions, and the BD CellQuest Pro software was used for analysis.

### Animal Tumor Model

BALB/c nude female mice (5–6 weeks old) raised in a specific animal facility were used to construct a xenograft model. HCC1937-FR cells (1 × 10^7^) suspended in 0.2 ml of PBS were inoculated subcutaneously into the backs of the nude mice. Mouse xenograft models were randomly divided into 4 groups and treated for 21 days: fluzoparib (25 mg/kg/bid), chidamide (5 mg/kg/bw), combined (fluzoparib 25 mg/kg/bid + chidamide 5 mg/kg/bw), and controls without any drug treatment. Xenograft weight and size were measured every 3 days. Tumor volume was calculated according to the formula: V = (length × width^2^)/2. All animal experiments conformed to the requirements of our institutional ethics committee.

### Western Blotting

Cells were treated with the experimental drugs for 48 h, after which their cytoplasm and nuclear protein was extracted (Cowin Bio., Beijing, China). Equal amounts of protein, processed using 12% SDS-PAGE (Cowin Bio., Beijing, China), were transferred to the PVDF membrane. The membrane, after blocking with 5% skim milk for 2 h, was incubated with a primary antibody at 4°C overnight. After rewarming the next day, the membrane was incubated with a secondary antibody at 37°C for 1 h. The Tanon 2500 chemiluminescence imaging system (Tanon, China) was used to detect the membranes. Further density and quantitative analyses were performed using Image J software.

### Cell Transfection

Si-RAD51, si-MRE11, and si-NC vectors for cell transfection were synthesized by Biological Company GenePharma (Shanghai, China). Fluzoparib-resistant cells were cultured in 6-well plastic plates until they reached 80% confluence. Transfection was performed using Lipofectamine 3000 as instructed by the manufacturer.

### Quantitative Real-Time Polymerase Chain Reaction

Intracellular mRNA was extracted using the AxyPrep mRNA Small Preparation Kit. cDNA was created *via* reverse transcription using HiScript III RT SuperMix for qPCR(+Gdna wiper). Reverse transcription was performed at 37°C for 15 min and 85°C for 5 s. Quantitative RT-PCR was performed using the ChamQ Universal SYBR qPCR Master Mix (Vazyme, Nanjing, China). Response conditions were as follows: 3 min at 95°C and then 10 s at 95°C for 40 cycles and 3 min at 95°C. mRNA relative expression levels were calculated using the 2^−△△Ct^ method. The primers are listed in **Table 1**.

**Table 1:** Primers in this study.

	Sequence (5′-3′)	Usage
RAD51F	CAACACAGACCACCAGACCC	qRT-PCR
RAD51R	AGAAGCATCCGCAGAAACCT	qRT-PCR
MRE11F	TCAGATCTCAGTCAGAGGAGTC	qRT-PCR
MRE11R	AGCCATCTGTTCTGCTAAATCT	qRT-PCR
GAPDHF	ACCACAGTCCATGCCATCAC	qRT-PCR
GAPDHR	TCCACCACCCTGTTGCTGTA	qRT-PCR
Si-RAD51	GCCCUUUACAGAACAGACUTT	Knockdown
	AGUCUGUUCUGUAAAGGGCTT	
Si-MRE11	GGCCUGUCCAGUUUGAAAUTT	Knockdown
	AUUUCAAACUGGACAGGCCTT	
NC sense	UUCUCCGAACGUGUCACGUTT	Knockdown
NC antisense	ACGUGACACGUUCGGAGAATT	Knockdown

### Statistical Analysis

Statistical analyses utilized the IBM SPSS 23.0 software (Armonk, NY, USA). Data statistics were expressed as mean ± SD. One-way analysis of variance (ANOVA) was used to measure statistically significant differences between the different experimental groups. *p* < 0.05 was considered statistically significant.

## Results

### HCC1937-FR and MDA-MB-468-FR Fluzoparib Resistance

**Figure 1:**
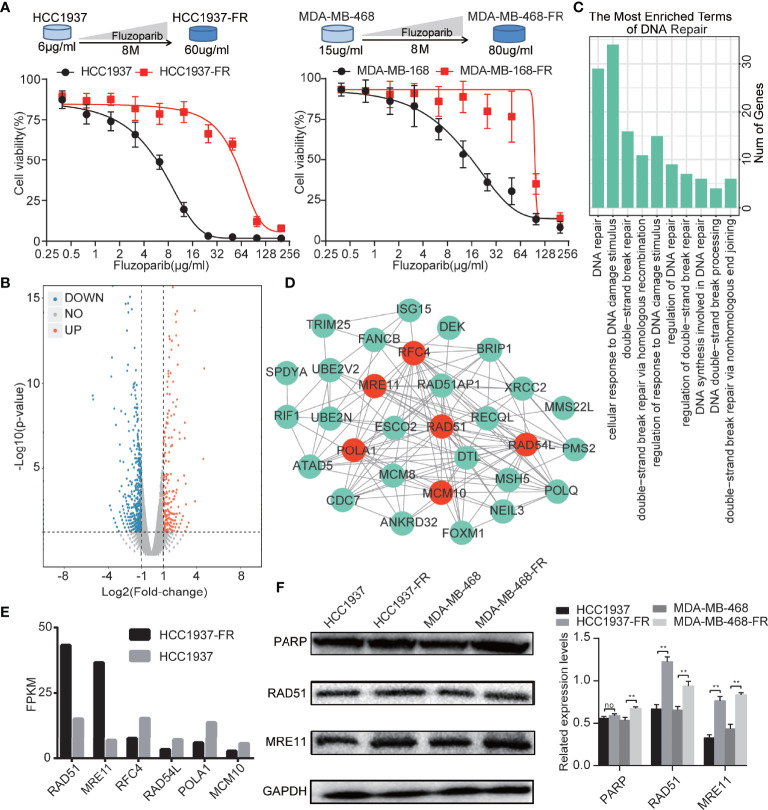
Generation of TNBC cells with acquired resistance to fluzoparib and the identification of key drug resistance genes. **(A)** Schematic plot of the construction of the fluzoparib-resistant HCC1937 and MDA-MB-468 cell lines. Dose-response curves of parental and fluzoparib-resistant HCC1937 and MDA-MB-468 cells treated with different concentrations of fluzoparib for 48 h. **(B)** Volcano plot of the differential gene analysis of parental and fluzoparib-resistant HCC1937 cells *via* gene sequencing. Red dots represent upregulated genes, and blue dots represent downregulated genes. **(C)** Enrichment analysis related to DNA damage repair. **(D)** Construction of a protein–protein interaction network. The hub drug resistance genes represented by red dots had the highest gene association in the network. **(E)** Gene expression levels of hub drug resistance genes in parental and fluzoparib-resistant HCC1937 cells. **(F)** Immunoblots of RAD51, MRE11, and PARP in parental and fluzoparib-resistant cells. Data are written as mean ± SD of three independent experiments. ^**^ *p* < 0.01 compared with the parental cell group. No, no significance.

To evaluate the cytotoxicity of fluzoparib on parental and drug-resistant cell lines, MTT assays were performed to test cell viability after exposure to various concentrations of fluzoparib for 48 h. As shown in **Figure 1A**, with increased fluzoparib concentrations, the growth of parental and drug-resistant cells was significantly reduced and the cell viability of drug-resistant cell lines was significantly higher than that of parental cell lines. The mean IC_50_ values of fluzoparib for HCC1937, MDA-MB-468, HCC1937-FR, and MDA-MB-468-FR cells were 6, 15, 60, and 80 μg/ml, respectively. The resistance indices of HCC1937-FR and MDA-MB-468-FR were 10 and 5.33, respectively.

### Identification and Biological Function Analysis of DEGs in Drug-Resistant Cells

As shown in the volcano plot (**Figure 1B**), a total of 616 DEGs were developed using high-throughput sequencing, including 393 DEG encoding proteins. When these DEG encoding proteins were uploaded to the STRING database for enrichment analysis, as shown in **Figure 1C**, 95 biological process terms were returned, of which 10 involved DNA damage repair, DNA repair, cellular response to DNA damage, double-strand break repair, double-strand break repair *via* homologous recombination, regulation of response to DNA damage stimulus, regulation of DNA repair, regulation of double-strand break repair, DNA synthesis involved in DNA repair, DNA double-strand break processing, and double-strand break repair *via* nonhomologous end joining. A total of 37 DEGs identified by the enrichment analysis terms were related to DNA damage repair.

### Identification of Hub Drug Resistance Genes

The 37 DEGs involved in DNA damage repair were uploaded to the STRING database to analyze their protein–protein interaction network. Visual analysis was performed using Cytoscape. The top 6 genes with the highest gene association (degree ≧14) were defined as hub drug resistance genes. As shown in **Figure 1D**, 6 hub drug resistance genes were identified, including RAD51, MRE11, POLA1, RAD54L, RFC4, and MCM10. RAD51 and MRE11 were highly expressed in drug-resistant cells, while POLA1, RAD54L, RFC4, and MCM10 had lower levels of expression (**Figure 1E**). RAD51 and MRE11 were therefore selected for subsequent analysis. As shown in **Figure 1F**, HCC1937-FR and MDA-MB-468-FR had higher levels of RAD51 and MRE11 protein expression than parental controls.

### Determine the Optimal Dosage of Chidamide

**Figure 2:**
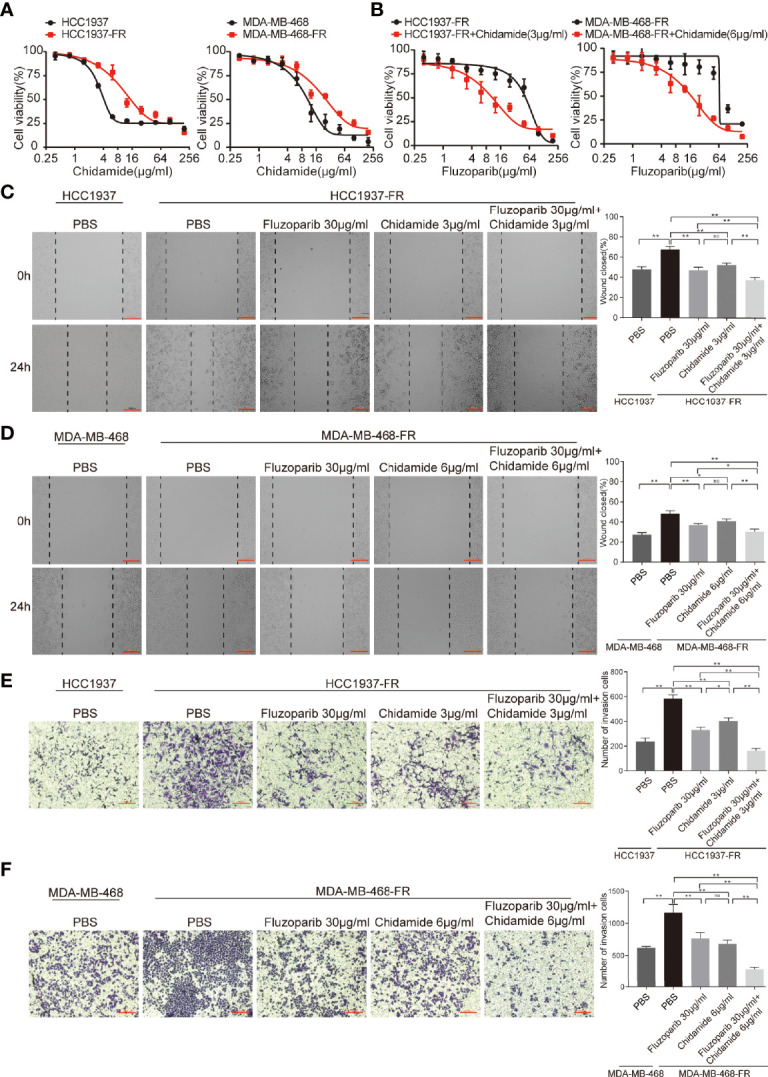
Effects of chidamide and fluzoparib on cell migration. **(A)** Dose-response curves of parental and fluzoparib-resistant cells treated with different concentrations of chidamide for 48 h. **(B)** Dose-response curves of parental and fluzoparib-resistant cells treated with different concentrations of fluzoparib combined with 3 or 6 μg/ml of chidamide. **(C, D)** Wound-healing assay to assess the effects of fluzoparib and chidamide on parental and fluzoparib-resistant HCC1937 and MDA-MB-468 cell migration ability. **(E, F)** Transwell invasion assays assessed the effects of fluzoparib and chidamide on parental and fluzoparib-resistant HCC1937 and MDA-MB-468 invasiveness. Data represent the mean ± SD of three independent experiments. ^*^ *p* < 0.05; ^**^ *p* < 0.01. No, no significance.

To assess the cytotoxicity of chidamide on parental and drug-resistant cell lines, cell viability was measured using MTT assays after exposure to various concentrations of chidamide for 48 h. As shown in **Figure 2A**, the survival rate of both parental and drug-resistant tumor cells gradually decreased with increased concentrations of chidamide. Results demonstrated that the inhibitory effects of chidamide on HCC1937 were better than on HCC1937-FR at a chidamide dose of ≥3 μg/ml, while at a dose ≥6 μg/ml, its inhibitory effect on MDA-MB-468 was better than on MDA-MB-468-FR. Doses of 3 and 6 μg/ml were therefore selected as subsequent experimental concentrations for HCC1937-FR and MDA-MB-468-FR, as at this concentration chidamide had little effect on their cell viability (cell viability was 85.9% and 85.2%, respectively).

### Chidamide Effectively Reverses the Fluzoparib-Resistance of Drug-Resistant Cells

To further evaluate the cytotoxicity of chidamide and fluzoparib on HCC1937-FR and MDA-MB-468-FR, cell viability was again detected using MTT assays after treatment with 3 or 6 μg/ml of chidamide combined with different concentrations of fluzoparib. As shown in **Figure 2B**, chidamide combined with fluzoparib significantly inhibited the proliferation of HCC1937-FR and MDA-MB-468-FR. After statistical analysis of tumor drug concentration-survival rate using SPSS software, the IC_50_ of HCC1937-FR to fluzoparib decreased from 60 to 9.6 μg/ml, while the IC_50_ of MDA-MB-468-FR to fluzoparib dropped from 80 to 20 μg/ml. The combined index of chidamide and fluzoparib was calculated using Compusyn software to determine if the combined effects of the two drugs had a coordinating effect. As shown in **Tables 2**, **3**, 3 μg/ml chidamide combined with ≥3.125 μg/ml fluzoparib had a good synergistic effect on the inhibition of HCC1937-FR cell proliferation, and the proliferation of MDA-MB-468-FR cells was effectively inhibited by ≥6 μg/ml chidamide combined with 12.5 μg/ml fluzoparib.

**Table 2:** The combination index of different doses of fluzoparib and 3 μg/ml chidamide: HCC1937-FR.

Chidamide (μg/ml)	Fluzoparib (μg/ml)	CI
3	1.5625	1.11351
3	3.125	0.73161
3	6.25	0.55963
3	12.5	0.47928
3	25	0.76496
3	50	0.51067
3	100	0.56623

**Table 3:** The combination index of different doses of fluzoparib and 6 μg/ml chidamide: MDA-MB-468-FR.

Chidamide (μg/ml)	Fluzoparib (μg/ml)	CI
6	6.25	4.12292
6	12.5	0.50483
6	25	0.60663
6	50	0.26538
6	100	0.18174
6	200	0.10877

### The Ability of Drug-Resistant Cells to Migrate and Invade Was Reduced

The migration ability of parental and drug-resistant cells and the effect of fluzoparib and chidamide on drug-resistant cell migration were measured using wound-healing assays. As shown in **Figures 2C, D**, the migration rates of the parental cells (HCC1937 and MDA-MB-468) were 47.98% ± 2.51% and 27.30% ± 2.08%, compared with 67.64% ± 3.10% and 48.17% ± 2.98% of the drug-resistant cells (HCC1937-FR and MDA-MB-468-FR), respectively (*p* < 0.01). After 24 h of treatment with fluzoparib and chidamide alone or in combination, the migration rate of HCC1937-FR cells was 46.88% ± 3.14% in the fluzoparib single agent, 51.94% ± 2.05% in the chidamide single agent, and 37.39% ± 2.34% in the combined group. Also, for MDA-MB-468-FR, the migration rate of cancer cells was 38.70% ± 3.15% in the fluzoparib single agent, 40.59% ± 2.34% in the chidamide single agent, and 30.32% ± 2.55% in the combined group. Fluzoparib combined with chidamide significantly inhibited the migration of HCC1937-FR and MDA-MB-468-FR cells (*p* < 0.01).

Similar observations were seen in the Transwell invasion assay. As shown in **Figures 2E, F**, compared with parental cells, HCC1937-FR and MDA-MB-468-FR cells significantly increased the number of cells that passed through the Transwell chamber, representing significantly enhanced invasiveness (*p* < 0.01). The numbers of invasive cells in the fluzoparib and chidamide groups were significantly decreased compared with controls, and the inhibitory effects of fluzoparib in combination with chidamide were more significant than the single drug groups (*p* < 0.01).

### The Antiapoptotic Ability of Drug-Resistant Cells Is Weakened by Antitumor Treatments

**Figure 3:**
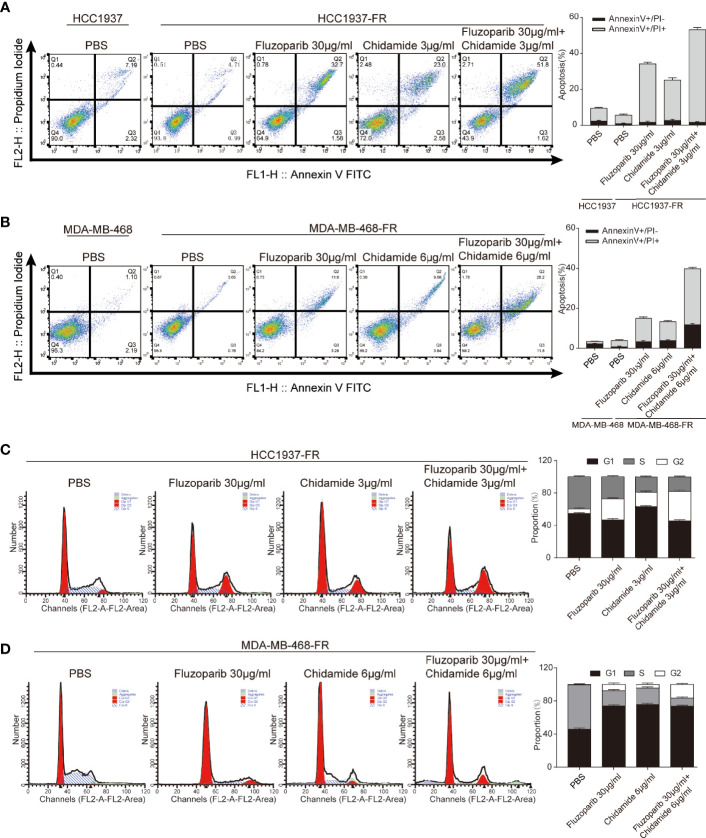
Effects of chidamide and fluzoparib on cell cycle and apoptosis. **(A, B)** Cell apoptosis was detected using Annexin V-FITC/PI double staining followed by flow cytometry for parental and fluzoparib-resistant HCC1937 and MDA-MB-468 cells after incubation with PBS, fluzoparib, chidamide, or a combination treatment (fluzoparib + chidamide) for 24 h. **(C, D)** Cell cycle analysis using PI staining and following flow cytometry for HCC1937-FR and MDA-MB-468-FR cells after incubation with PBS, fluzoparib, chidamide, or a combination treatment (fluzoparib + chidamide) for 24 h. Data represent the mean ± SD of three independent experiments.

Flow cytometry was used to measure the apoptosis rate and cell cycle of parental and drug-resistant cells. Results are shown in **Figures 3A, B**. The apoptosis rates of HCC1937 and MDA-MB-468 cells were 9.51% and 3.29%, respectively, while those of HCC1937-FR and MDA-MB-468-FR cells were 5.7% and 3.81%, respectively. The antiapoptosis rate of HCC1937-FR cells was significantly higher than that of parental cells (*p* < 0.05), but there was no significant difference in the apoptosis rates of MDA-MB-468-FR and MDA-MB-468. The apoptosis rates of HCC1937-FR cells after fluzoparib single agent, chidamide single agent, and combination exposure were 34.28%, 25.58%, and 53.42%, respectively. The apoptosis rates in the single drug groups were significantly higher than that of the control group (*p* < 0.05). The apoptosis rate of the combined group was also significantly higher than those of the single drug cell groups (*p* < 0.05). The apoptosis rates of MDA-MB-468-FR after fluzoparib single agent, chidamide single agent, and combination exposure were 15.08%, 13.52%, and 40%, respectively. These results were also statistically significant (*p* < 0.05). The cell cycle distributions of HCC1937-FR and MDA-MB-468-FR cells after drug treatment are shown in **Figures 3C, D**. Single drug groups prolonged the G2/M phase of the drug-resistant cells, while the combination group exerted a greater effect at the G2/M phase (*p* < 0.05).

### *In Vivo* Anticancer Effects of Fluzoparib and Chidamide in HCC1937-FR Breast Cancer Xenograft Models

**Figure 4:**
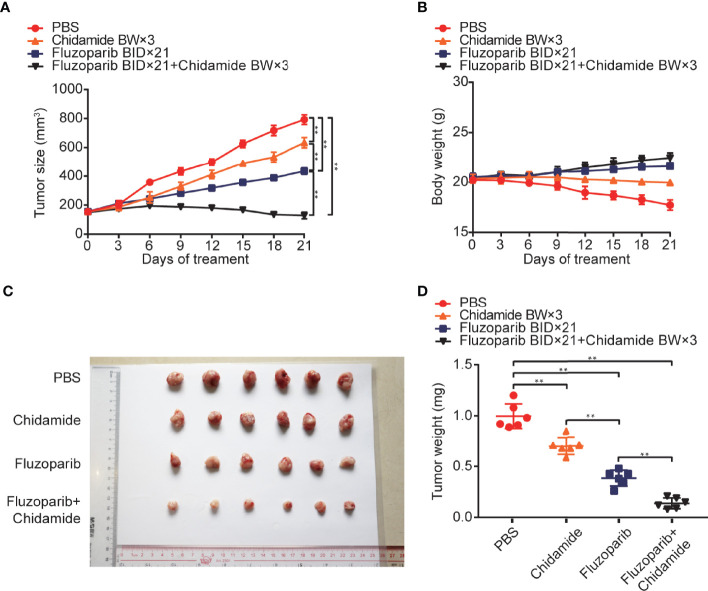
*In vivo* anticancer effects of fluzoparib and chidamide in fluzoparib-resistant HCC1937 breast cancer xenograft models. Randomly grouped nude mice were treated with PBS, fluzoparib (25 mg/kg/bid), chidamide (5 mg/kg/bw), or a combination treatment (fluzoparib 25 mg/kg/bid + chidamide 5 mg/kg/bw) for 21 days. **(A, B)** Tumor growth ratio curve and body weight change every 3 days after the onset of treatment. **(C, D)** Photographs of the exfoliated tumors and weight obtained on day 21 of treatment. ^**^ *p* < 0.01.

As shown in **Figure 4**, while both fluzoparib and chidamide inhibited HCC1937-FR breast cancer growth (*p* < 0.05), the combination of these drugs more significantly inhibited neoplasm growth (*p* < 0.05). No general toxicity was observed as no weight loss occurred in any treatment group (*p* < 0.05).

### Molecular Mechanism of Chidamide Reversing Fluzoparib Resistance

**Figure 5:**
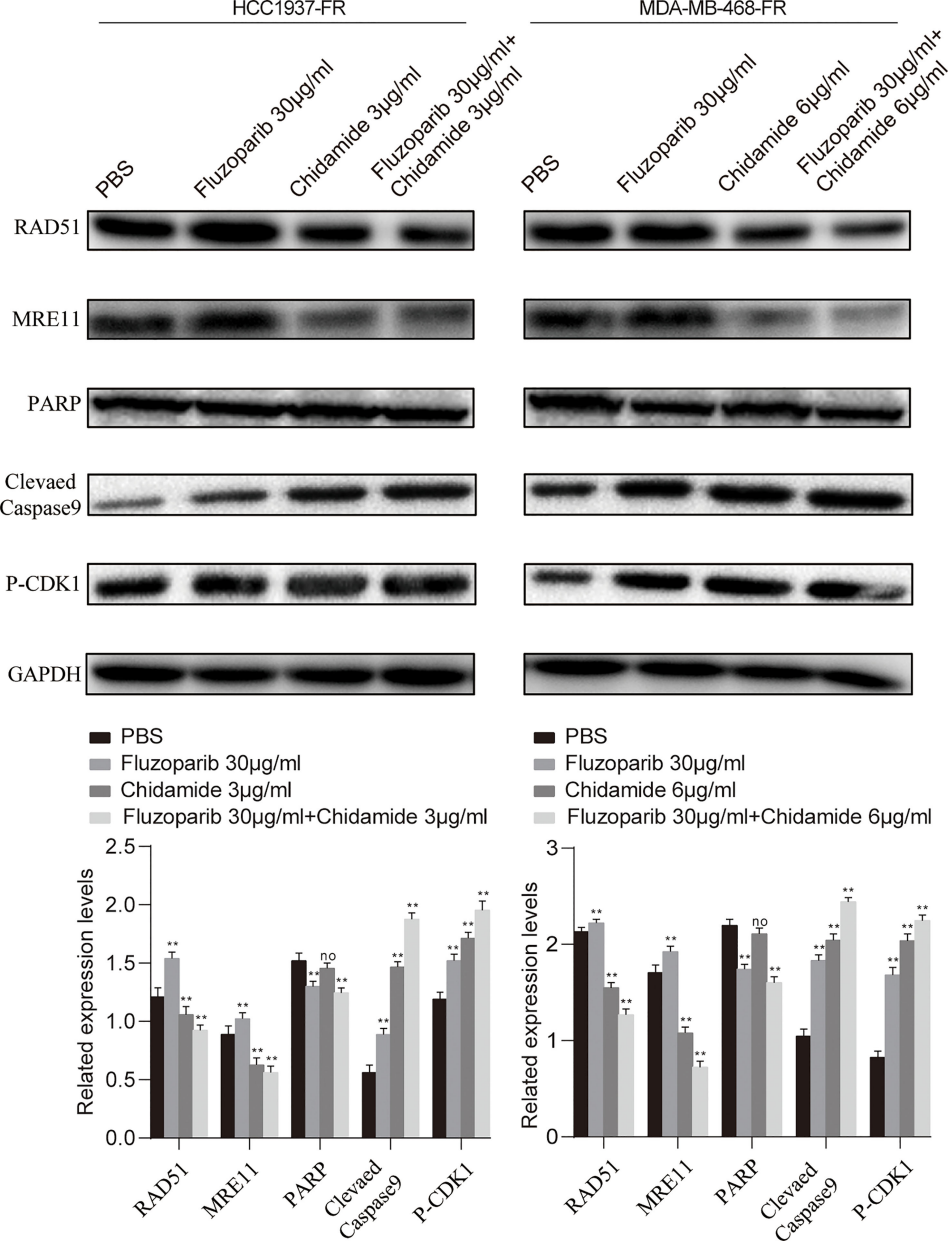
Molecular mechanism studies of fluzoparib-resistant HCC1937 and MDA-MB-468 cells after treatment with PBS, fluzoparib, chidamide, or a combination treatment (fluzoparib + chidamide). Immunoblot analysis of RAD51, MRE11, PARP, BCL-XL, and P-CDK1 and quantitative analysis. Data represent the mean ± SD of three independent experiments. ^**^ *p* < 0.05 compared with the PBS group. No, no significance. **p < 0.01.

As shown in **Figure 5**, fluzoparib significantly reduced PARP protein expression (*p* < 0.05), while chidamide alone or combined with fluzoparib did not affect PARP expression. The combined effect of the two drugs significantly reduced the expression of the RAD51 and MRE11 proteins in drug-resistant cells. The results of cycle and apoptosis assays showed that the drugs block the cell cycle in the G2/M phase and induce apoptosis. These results suggest that fluzoparib combined with chidamide significantly increased the expression levels of P-CDK1 and cleaved Caspase9.

### Genetic Suppression of RAD51 and MRE11 Enhances the Sensitivity of Drug-Resistant Cells to Fluzoparib

**Figure 6:**
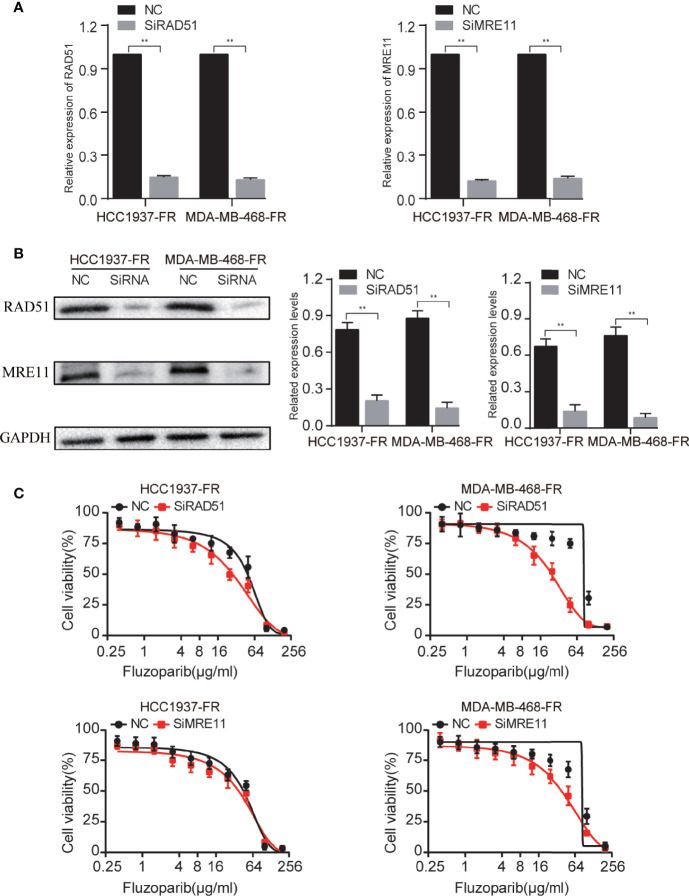
Genetic suppression of RAD51 and MRE11 enhances the sensitivity of drug-resistant cells to fluzoparib. **(A)** qRT-PCR was used to detect RAD51 and MRE11 after transfection with si-RAD51, si-MRE11, and NC in fluzoparib-resistant cells. **(B)** Immunoblots of RAD51 and MRE11 in fluzoparib-resistant cells transfected with si-RAD51, si-MRE11, and NC. **(C)** Dose response of fluzoparib-resistant cells treated with fluzoparib after transfection with si-RAD51, si-MRE11, and NC. NC, negative control. ^**^ *p* < 0.01.

Compared with negative controls (NC), the expression of the mRNA and protein of RAD51 and MRE11 were significantly decreased (**Figures 6A, B** **)**. To further study the response of transfected cells to fluzoparib, an MTT assay was used to detect the cell viability of HCC1937-FR and MDA-MB-468-FR cells transfected for 24 h. As shown in **Figure 6C**, the proliferation of transfected cells was significantly inhibited compared with the control group with increased drug concentrations. These data suggest that knockdown of the RAD51 and MRE11 genes could enhance the sensitivity of HCC1937-FR and MDA-MB-468-FR cells to fluzoparib.

## Discussion

The increasing incidence of breast cancer in women is a major women’s health problem. First-line treatment options for breast cancer include chemotherapy, hormones, and targeted therapy (37). Immunotherapy drugs have also recently shown promise (38). TNBC, which accounts for approximately 15% of all breast cancers, is insensitive to endocrine and molecular-targeted drugs (39). Precisely targeted therapeutic PARPi have achieved promising results in clinical trials by inhibiting PARP enzyme function and hindering the possibility of DNA repair in tumor cells, thereby accelerating tumor cell death (40). A series of PARP-targeted drugs have been developed, including olaparib, talazoparib, and fluzoparib.

OlympiAD, a randomized, open-label, and phase III trial, evaluated olaparib monotherapy versus a standard chemotherapy regimen (41), reporting that olaparib prolonged PFS from 4.2 to 7.0 months, significantly reduced the risk of disease progression by 42% and was well tolerated (42). EMBRACA, an open-label phase III trial, reported that talazoparib significantly prolonged PFS and reduced the risk of disease progression, and that the objective response rate of 62.6% in the talazoparib group was more than double that of the chemotherapy group (27.2%) (*p* < 0.0001) (43). Fluzoparib, a synthetic derivative based on olaparib, exhibits antitumor activity against breast cancer as a single agent in a phase 1 study in advanced solid tumors and has a significant antitumor efficacy in combination with apatinib or apatinib and paclitaxel, without extra toxicity (44). Chemotherapy resistance reflects the strong adaptability of tumor cells, so it is important to further explore how to avoid PARPi resistance and develop promising therapeutic strategies. Given the excellent antitumor effects of chidamide against DNA damage repair, we performed this study to determine if chidamide could reverse fluzoparib resistance.

In this study, we constructed the fluzoparib-resistant triple-negative breast cancer cell lines HCC1937-FR and MDA-MB-468-FR, and further studied the changes in the biological functions of these resistant cells. *In vitro* experiments confirmed that the proliferation, migration, and invasiveness of drug-resistant cells were enhanced compared with parental cells, and that their degree of virility was also increased. Similar studies showed that the migration ability of HCC1937 cells resistant to talazoparib was also enhanced (45). We further investigated the effects of chidamide and fluzoparib on drug-resistant cell lines and xenograft models. MTT and flow cytometry results showed that chidamide combined with fluzoparib could significantly inhibit the proliferation of drug-resistant cells and reduce the IC_50_ of fluzoparib. In addition, both chidamide and fluzoparib exhibited certain inhibitory effects on the migration and invasiveness of the drug-resistant cells and, more significantly, the inhibitory effects of the combination of these two drugs were more obvious. Although previous studies have shown that PARPi and HDACi can arrest triple-negative breast cancer cells in the G2/M phase, no works have evaluated the effect of PARPi and HDACi on the drug-resistant cell cycle (32). In the present study, cycle results showed that all single drugs could arrest drug-resistant cells in the G2/M phase and that the combined group had a more significant effect. Xenograft model results demonstrated that the antitumor treatment effects of the combination groups were greater than any other single drug group, which matched our *in vitro* findings. The nude mice in all treatment groups also did not show significant weight loss.

**Figure 7:**
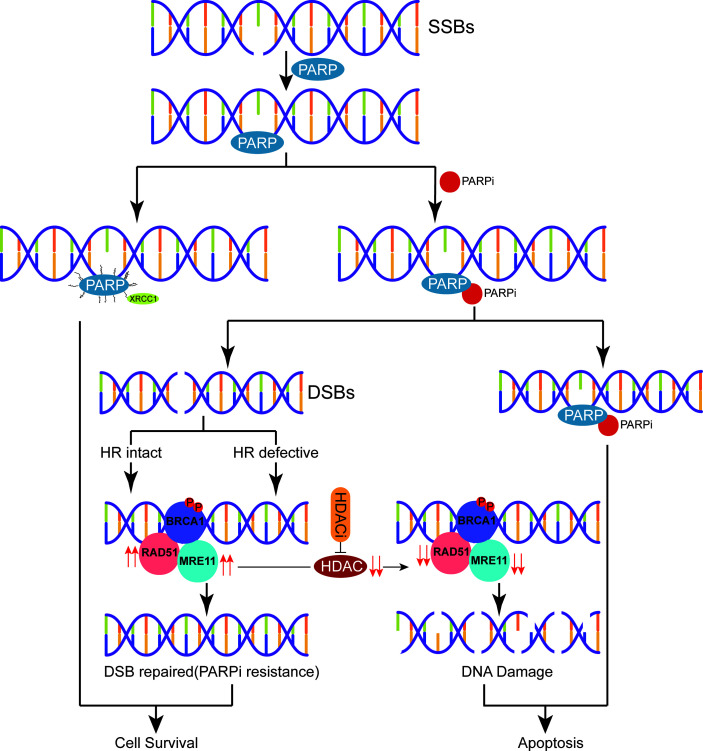
A proposed schematic of PARPi resistance in triple-negative breast cancer. After DNA single-strand breaks (SSBs) occur, PARP binds with the damaged site and recruits related DNA repair proteins to repair the damaged DNA. PARPi can be combined with PARP to inhibit SSB repair leading to apoptosis or process DNA SSBs and initiate homologous recombination (HR) repair. Due to the relative overexpression of the HR repair-related proteins RAD51 and MRE11 in the setting of drug resistance or reduced sensitivity to PARPi, HDACi perpetuates DNA damage by regulating the expression of RAD51 and MRE11, eventually leading to cell apoptosis.

We performed transcriptome sequencing on drug-resistant and parental cells, identifying DEGs using differential analysis and selecting hub drug-resistant genes related to DNA damage repair. To further explore the potential molecular mechanisms and signaling pathways of chidamide reversal of fluzoparib resistance, we used Western blotting (WB) to evaluate molecular changes after drug exposure. We found that both the gene and protein levels of RAD51 and MRE1 were highly expressed in drug-resistant cells, and that their protein levels were significantly downregulated after treatment with chidamide combined with fluzoparib. Min et al. (33) confirmed that HDACi combined with olaparib could downregulate the expression of RAD51 and MRE11 in TNBC. Furthermore, by knocking out the RAD51 and MRE11 genes in fluzoparib-resistant cell lines, transfected cells had enhanced sensitivity to fluzoparib-treated cells. However, the inhibitory effects of RAD51 and MRE11 gene suppression on the proliferation of drug-resistant cell lines were not as significant as that of chidamide. The reason may be that HDACi may affect multiple DNA damage repair proteins at the same time or impact the interactions between HDAC and DNA damage repair proteins. These phenomena suggest that RAD51 and MRE11, as key drug resistance genes, are significantly related to fluzoparib resistance in TNBC cells. RAD51, a key regulator of DNA fidelity, is involved in cell cycle regulation, repair of homologous recombination, and replication stress response, which are essential for the stability of the genome (46). Human RAD51 has DNA-dependent ATPase activity and performs DNA repair and recombination through homologous pairing and strand exchange between DNA molecules (47). In breast cancer, high expression of RAD51 has been associated with cancer cell metastasis, tumor chemotherapy resistance, and tumor radiotherapy insensitivity (48). MRE11, a nuclear protein, participates in homologous recombination and telomere length maintenance, and has 3′ to 5′ exonuclease and endonuclease activity (49). MRE11 can form an MRX/MRN complex with RAD50 homologues, which depends on the activity of nucleases to participate in DNA homologous recombination repair (50). Studies have shown that the functional defects and low expression of MRX/MRN and its components were associated with an increased tendency towards sustained DNA damage, cell instability, and malignant transformation and can also affect the sensitivity of cancer cells to chemotherapy and radiotherapy (51). HDACi could downregulate DNA damage repair proteins. The potential mechanism for this may be that HDACi induces proteasomal deterioration of homologous recombination repair-related proteins, or that HDACi reduces E2F1 binding to the promoters of BRCA1, CHK1, and RAD51, thereby reducing the transcription of these genes (40, 52). The elevated levels of the key regulatory proteins P-CDK1 and cleaved Caspase9 again confirm that the combination of fluzoparib and chidamide can block the cell cycle in the G2/M phase and promote the apoptosis of drug-resistant cells. The present work suggests that the drug resistance or decreased sensitivity of TNBC cells to PARPi is caused by the relative overexpression of the DNA damage repair-related proteins RAD51 and MRE11, while HDACi caused persistent DNA damage by downregulating RAD51 and MRE11, eventually leading to cell apoptosis. As a result, we have proposed a model depicting the molecular mechanisms of chidamide reversal of fluzoparib resistance (**Figure 7**). There are many kinds of HDAC and PARP inhibitors that are currently available, so the application of these two drugs against drug resistance still requires further study.

In conclusion, this is the first study to provide evidence of PARPi resistance reversal by HDACi *in vivo* and *in vitro* and to propose the molecular mechanism behind the reversal of resistance, providing guidance for breast cancer treatment.

## Data Availability Statement

The original contributions presented in the study are included in the article/**Supplementary Materials**. Further inquiries can be directed to the corresponding author.

## Ethics Statement

The animal study was reviewed and approved by the Ethics Committee of the First Affiliated Hospital of Henan University of Science and Technology.

## Author Contributions

Conception and design: XW and XL. Data acquisition and analysis: ZW, JL, and XY. Writing original draft: XL. Writing review and editing: YW, LW, and YL. Data visualization: YL and XL. Supervision: XW and YL. The authors read and approved the final manuscript.

## Funding

This work was supported in part by Medical Science and Technology project grants of Henan Province (LHGJ20200576).

## Conflict of Interest

The authors declare that the research was conducted in the absence of any commercial or financial relationships that could be construed as a potential conflict of interest.

## Publisher’s Note

All claims expressed in this article are solely those of the authors and do not necessarily represent those of their affiliated organizations, or those of the publisher, the editors and the reviewers. Any product that may be evaluated in this article, or claim that may be made by its manufacturer, is not guaranteed or endorsed by the publisher.
