# Supplementary material for: Chidamide Reverses Fluzoparib Resistance in Triple-Negative Breast Cancer Cells
Source: Front Oncol. 2022 Feb 18;12:819714. doi: 10.3389/fonc.2022.819714 (PMC8894594; doi:10.3389/fonc.2022.819714)
Supplement: Supplementary file 7 [file Table_5.docx]

a1<-read.csv("F:/RyuyanYUYING/Acexu/CEXU/1111111.csv", header=T) names(data)

ggplot(data=data, aes(x=term.description,y=observed.gene.count, fill=category)) + geom_bar(stat="identity", width=0.8)

library(ggplot2)

GO_term_order=factor(as.integer(rownames(data)),labels=data$term.description)

#66C3A5 #FD8D62 #66C3A5

COLS <- c("#66C3A5", "#FD8D62", "#FD8D62")

ggplot(data=data1, aes(x=GO_term_order,y=observed.gene.count, fill=category)) +

geom_bar(stat="identity", width=0.8) +

scale_fill_manual(values = COLS) + theme_bw() + coord_flip() +

xlab("GO term") + ylab("Num of Genes") +labs(title = "The Most Enriched GO Terms")+

theme(axis.text.x=element_text(face = "bold", color="gray50",angle = 0,vjust = 1, hjust = 1))+

theme(axis.text.y=element_text(size=6))
